# Substrate mapping of the left atrium in persistent atrial fibrillation: spatial correlation of localized complex conduction patterns in global charge-density maps to low-voltage areas in 3D contact bipolar voltage maps

**DOI:** 10.1007/s10840-020-00926-4

**Published:** 2021-01-08

**Authors:** Gian-Battista Chierchia, Juan Sieira, Annelies Vanderper, Thiago Guimarães Osorio, Gezim Bala, Erwin Stroker, Pedro Brugada, Maysam Al Houssari, Federico Cecchini, Joerelle Mojica, Ingrid Overeinder, Antonio Bisignani, Vincenzo Mitraglia, Serge Boveda, Gaetano Paparella, Carlo de Asmundis

**Affiliations:** Heart Rhythm Management Centre, Postgraduate Program in Cardiac Electrophysiology and Pacing, European Reference Networks Guard-Heart, Universitair Ziekenhuis Brussel, Vrije Universiteit Brussel, Laarbeeklaan 101, Brussels, Belgium

**Keywords:** Substrate mapping, Dipole density noncontact mapping, SuperMap algorithm, Atrial fibrillation, Low-voltage areas, 3D-mapping

## Abstract

**Purpose:**

This study aimed to investigate the spatial relationship between low-voltage areas (LVAs) in bipolar voltage mapping (BVM) and localized complex conduction (LCC)-cores in a global, non-contact, charge-density-based imaging, and mapping system (AcM).

**Methods:**

Patients with history of index PVI for PsAF and scheduled for a repeat ablation procedure for recurrence of the same arrhythmia were enrolled between August 2018 and February 2020. All patients underwent both substrate mappings of the left atrium (LA) with the CARTO 3D map-ping system and with AcM.

**Results:**

Ten patients where included in our analysis. All presented with persistency of PVI in all veins at the moment of repeat procedure. There was no linear relationship in BVM maps between SR and CSd (correlation coefficient 0.31 ± 0.15), SR and CSp (0.36 ± 0.12) and CSd and CSp (0.43 ± 0.10). The % overlap of localized irregular activation (LIA), localized rotational activation (LRA) and Focal (F) regions with LVA was lower at 0.2 mV compared to 0.5 mV (4.97 ± 7.39%, 3.27 ± 5.25%, 1.09 ± 1.92% and 12.59 ± 11.81%, 7.8 ± 9.20%, 4.62 ± 5.27%). Sensitivity and specificity are not significantly different when comparing composite maps with different LVA cut-offs. AURC was 0.46, 0.48, and 0.39 for LIA, LRA, and Focal, respectively.

**Conclusion:**

Due to wave front direction dependency, LVAs mapped with BVM in sinus rhythm and during coronary sinus pacing only partially overlap in patients with PsAF. LCC-cores mapped during PsAF partially co-localize with LVAs.

## Introduction

Pulmonary vein isolation is the cornerstone of AF ablation today [1]. Catheter ablation is currently associated with suboptimal outcome in patients with persistent atrial fibrillation (PsAF). Although PVI can guarantee freedom from PsAF in a substantial proportion of patients, up to 50% will experience an arrhythmia relapse within a year from the index procedure [2]. These relatively poor outcomes partially result from a lack in understanding of the mechanisms of PsAF and from the difficulty to create completely transmural and durable ablation lesions. These considerations have motivated many investigators to search for additional ablation strategies [3–5]. Substrate mapping and localization of low-voltage areas are increasingly being performed, worldwide [6]. The most common approach for characterizing the left atrial substrate is bipolar voltage mapping (BVM). Abnormally-low bipolar voltage amplitude is a putative marker of arrhythmogenic substrate [7]. Targeting low-voltage areas (LVAs) identified by BVM is currently proposed as a patient-specific tailored approach for the treatment of PsAF beyond PVI. Present day sequential 3D mapping systems are limited for this approach by temporal and spatial constraints. Furthermore, BV amplitude is dependent on rate and directionality of the conduction wave front [8]. Application of a global, non-contact, charge-density-based imaging, and mapping system (AcQMap, Acutus Medical, Carlsbad CA) (AcM) in PsAF has led to the identification of distinct localized complex conduction (LCC) patterns that may be relevant in the initiation and maintenance of PsAF [9]. These patterns have been classified as focal, characterized by early activation that did not generate from previous cardiac wavefronts; localized rotational activation (LRA) patterns, characterized by wavefront propagation rotating around a central obstacle; and localized irregular activity (LIA) in which the activation pattern displays repetitive, multidirectional, entry, exit, and pivoting conduction through and around a confined isthmus-like zone. To the best of our knowledge, little is known about the spatial correlation between LVA and LCC-cores. This study aimed to investigate the spatial relationship between LVAs in BVM and LCC-cores in AcM.

## Methods

### Patient population

Patients with history of index PVI for PsAF and scheduled for a repeat ablation procedure for recurrence of the same arrhythmia were enrolled between August 2018 and February 2020. All index procedures for AF are performed as PVI with the cryoballoon in our center. All patients underwent both substrate mappings of the left atrium (LA) with the CARTO 3D mapping system (Biosense Webster Inc., Diamond Bar, CA, USA) and with AcM. Patients in which (1) direct-current cardioversion (DCCV) could not restore SR, (2) SR could not be maintained during mapping, (3) pacing during bipolar voltage mapping induced AF or (4) presented with one or more reconnected veins were excluded from the study. The study protocol was carried out in accordance with the ethical principles for medical research involving human subjects established by the Declaration of Helsinki and was approved by the local ethics committee of our Institution. All patients provided written informed consent to the ablation procedure.

### Conventional 3D mapping: dynamic substrate mapping

Through a single transseptal access, a multi-splined mapping catheter (PentaRay catheter, Biosense Webster, Diamond Bar, CA) was positioned in the LA. High-density BVMs were sequentially generated on the CARTO 3D mapping system while roving the PentaRay catheter throughout the LA, first in SR and then during pacing from the distal and proximal coronary sinus (CSd and CSp) at 600 msec. Ultimately, three unique BVMs were acquired from each patient for comparative analysis between LVAs identified from BVMs with LCC-cores identified by AcM.

### Charge density global mapping

**Fig. 1:**
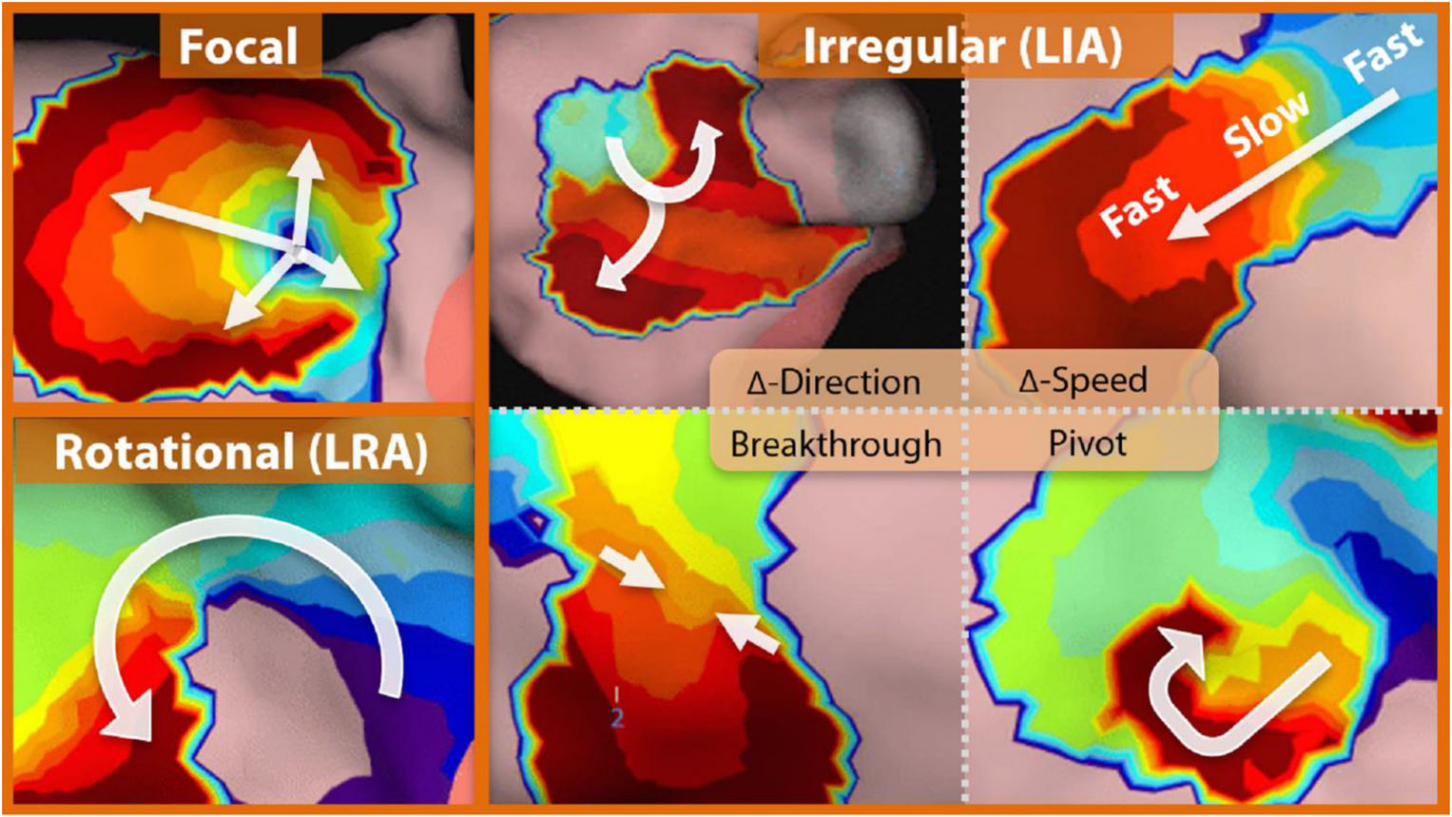
Commonly observed localized complex conduction patterns during atrial fibrillation

Activation maps of PsAF were acquired by AcM, previously described in detail by Shi et al. [9]. In summary, AcM provides maps of electrical activation across an ultrasound-acquired cardiac chamber surface. The AcQMap catheter (Acutus Medical, Carlsbad, CA) and system are designed to acquire data without the need to contact the chamber surface, which is referred to as “noncontact” mapping. The spatial resolution of the AQc map is 2 mm. The activation wave front can be displayed in its rawest form as either a charge- or voltage-based map of depolarization. A propagation-history map uses bands of color to show the location and velocity of the leading edge of the wave front over a set duration of time. Figure 1 represents the LCC patterns observed during PsAF. These LCC patterns are automatically detected and quantified using AcQTrack, an algorithm integrated into AcM methodology. The LIA algorithm computes the difference in angle and speed between cardiac conduction entering and leaving a confined region. Activations are grouped into entering and leaving the region based on the activation time with comparison to a central vertex. Mean conduction velocity vectors entering and leaving the region are then computed. If the angle-difference between entrance and exit vectors exceeds 90°, LIA is detected. The LRA algorithm computes the degrees of conduction propagation around a central point by summing the angle differences of sequential conduction velocity vector directions around the central point. If the rotational angle of conduction contiguously exceeds 270°, rotation is detected at the central point. Finally, the Focal algorithm determines whether an activation at a vertex came from a previous cardiac wavefront, arriving from adjacent vertices, or whether activation spontaneously started from the local vertex. Focal activation is detected at a vertex if an activation is earlier than its neighbors’ activation by at least 3 ms, and conduction spreads radially outward from the early activation.

### Procedural sequence

After femoral access, a transseptal puncture was performed as previously described. Then the AcQMap catheter was positioned within the left atrium and a map in PsAF was performed as described above. AcQTrack was applied on the AF maps to identify LCC-cores. DCCV was performed. Under CARTO mapping system guidance, BVMs were acquired in SR and during pacing from CSp and CSd using the PentaRay catheter, as described above and abnormally low-voltage zones were identified.

### Point-by-point analysis

All cross-analysis of CARTO and AcM maps was performed in a custom, offline software analysis program. For all BVMs, LVAs were defined as the aggregation of all points with BV amplitude < 0.2 and 0.5 mV. The LVA as a percentage of the total atrial surface area was calculated. The percentage of spatial overlap of LVAs between different BVMs was calculated using a point-by-point (PBP) analysis. Average voltage was calculated for all BVMs. The PVs and mitral valve were excluded from all calculations of spatial overlap. Spatial registration of CARTO and AcM LA surfaces was performed based on the following steps: (1) three pairs of points were placed on both maps (between left pulmonary veins, between right pulmonary veins and one point at the base of atrium), (2) both surfaces were fitted based on algorithm that minimized the mutual distance between all three pairs of points, and (3) spatial fitting was refined based on algorithm that minimized the total distance between all vertices on both surfaces. Further analysis was based on composite maps generated from the three different BVMs made in SR and during pacing from CSd and CSp. Composite maps were made with low-voltage definitions ranging from < 0.2 to 1 mV, in steps of 0.1 mV. PBP analysis was based on all vertices of the AcM anatomic mesh. For each AcM vertex, the closest vertex on the CARTO surface mesh was found. This resulted in approximately 3600 pairs of points for each comparison. Outliers of LIA, LRA, and F pattern-occurrence on AcM maps were removed based on an adaptive cut-off value. For each LCC pattern, the cut-off value was adjusted to achieve a 20% drop from the total time the LCC was present throughout the recording. For each composite map, the percentage of spatial-overlap of LVAs with LCC-cores, as determined by AcQTrack, was calculated. To further characterize the geometric relationship, the minimum distance between the border of the composite LVAs and the center of most prevalent LCC patterns was determined with a distance measurement tool on the reconstructed anatomies. When the LCC-cores were spatially localized among two or more LVAs, an average minimum distance was calculated. PBP comparison of composite LVAs to AcQTrack-identified LCC-cores was analyzed.

### Statistical analysis

The Student’s *t* test was used for comparison of different groups. For each composite map, the PBP analysis was used to calculate sensitivity and specificity for LCC patterns, respectively. ROC curve was generated for subsequent calculation of AURC. Cohen’s Kappa coefficient was calculated to calculate the inter-rater reliability between AcQTrack-detected LCC-cores and LVAs determined from composite BVMs.

## Results

### Baseline characteristics

Twenty-four patients were scheduled for consecutive mapping of the LA with CARTO and AcM. Ten patients were eligible for the comparative analysis. The remaining 14 patients did not meet the requirements for inclusion. Specifically, 10 patients were excluded because of failure to maintain SR during the procedure, 3 because AF was repeatedly induced during pacing for bipolar mapping, and 1 patient because of failure to restore SR with cardioversion at the beginning of the procedure. All patients were in PsAF at the beginning of the procedure. Baseline population characteristics are shown in Table 1.

**Table 1:** Baseline population characteristics

Age (years)		67.40 ± 8.83
Male (female)		8 (2)
BMI		26.76
AF type	Paroxysmal	0
	Persistent	10
Duration of AF (months)		31.7 ± 31.5
Hypertension		2 (8)
Diabetes		0 (10)
Dyslipidemia		4 (6)
CAD		2 (8)
Stroke		0 (10)
LVEF		52.2 ± 8.3

*AF* atrial fibrillation; *CAD* coronary artery disease; *LVEF* left ventricular ejection fraction

### Bipolar voltage amplitude

The average number of points taken with the PentaRay was not significantly different among the three BVMs. On average 2650 ± 1011, 3012 ± 1509, and 2790 ± 110 points were taken in SR, CSd pacing, and CSp pacing, respectively. The average amplitude of BVMs acquired in SR was significantly higher than the average amplitude of BVMs acquired during CSd pacing (0.992 ± 0.297 vs. 0.742 ± 0.201 mV, *p* = 0.05). The average amplitude of BVMs acquired during CSd pacing was significantly lower than the average amplitude of BVMs acquired during CSp pacing (0.742 ± 0.201 vs. 0.883 ± 0.231 mV, *p* = 0.05). There was no significant difference in the average amplitude of BVM acquired during SR versus during CSp pacing (0.992 ± 0.297 vs. 0.883 ± 0.231 mV, *p* = 0.128).

### Percentage of low-voltage area and low-voltage area overlap between BVMs

The percentage of area covered by LVA was not significantly different between the three classifications of BVMs (SR, CSd, and CSp pacing) for both 0.2 and 0.5 mV cut-off, Table 2. The percentage of spatial overlap between the LVAs was not significantly different among comparative groups for both 0.2 and 0.5 mV cut-off (Table 2). Specifically, spatial overlap of LVAs was 25.38 ± 17.18 and 37.70 ± 15.03 at 0.2 mV and 0.5 mV cut-off values, respectively, when comparing SR with CSd pacing; 21.09 ± 16.43 and 35.20 ± 17.49 at 0.2 mV and 0.5 mV cut-off values, respectively, when comparing SR with CSp pacing; and 27.49 ± 18.06 and 35.91 ± 17.00 mV cut-off values, respectively, when comparing CSd and CSp pacing. The correlation coefficients for PBP analysis are comparable among the different map groups for both 0.2 and 0.5 mV cut-off (Table 2).

**Table 2:** Comparison of low-voltage areas detected with bipolar voltage mapping generated in sinus rhythm and during coronary sinus distal and coronary sinus proximal pacing at 600 ms

	Maps	Units		
LVA definition		mV	0.2	0.5
Area % LVA	SR	%	7.60 ± 4.89	23.36 ± 12.31
Area % LVA	CSd	%	11.21 ± 12.16	29.56 ± 18.09
Area % LVA	CSp	%	9.98 ± 6.91	27.65 ± 16.70
% Spatial overlap	SR-CSd	%	25.38 ± 17.18	37.70 ± 15.03
% Spatial overlap	SR-CSp	%	21.09 ± 16.43	35.20 ± 17.49
% Spatial overlap	CSd-CSp	%	27.49 ± 18.06	35.91 ± 17.00
Correlation coefficient	SR-CSd	–	0.31 ± 0.15	0.31 ± 0.15
Correlation coefficient	SR-CSp	–	0.36 ± 0.123	0.36 ± 0.123
Correlation coefficient	CSd-CSp	–	0.43 ± 0.10	0.43 ± 0.10

*LVA* low-voltage areas

### Percentage of spatial overlap between LVAs and LCC-cores

**Fig. 2:**
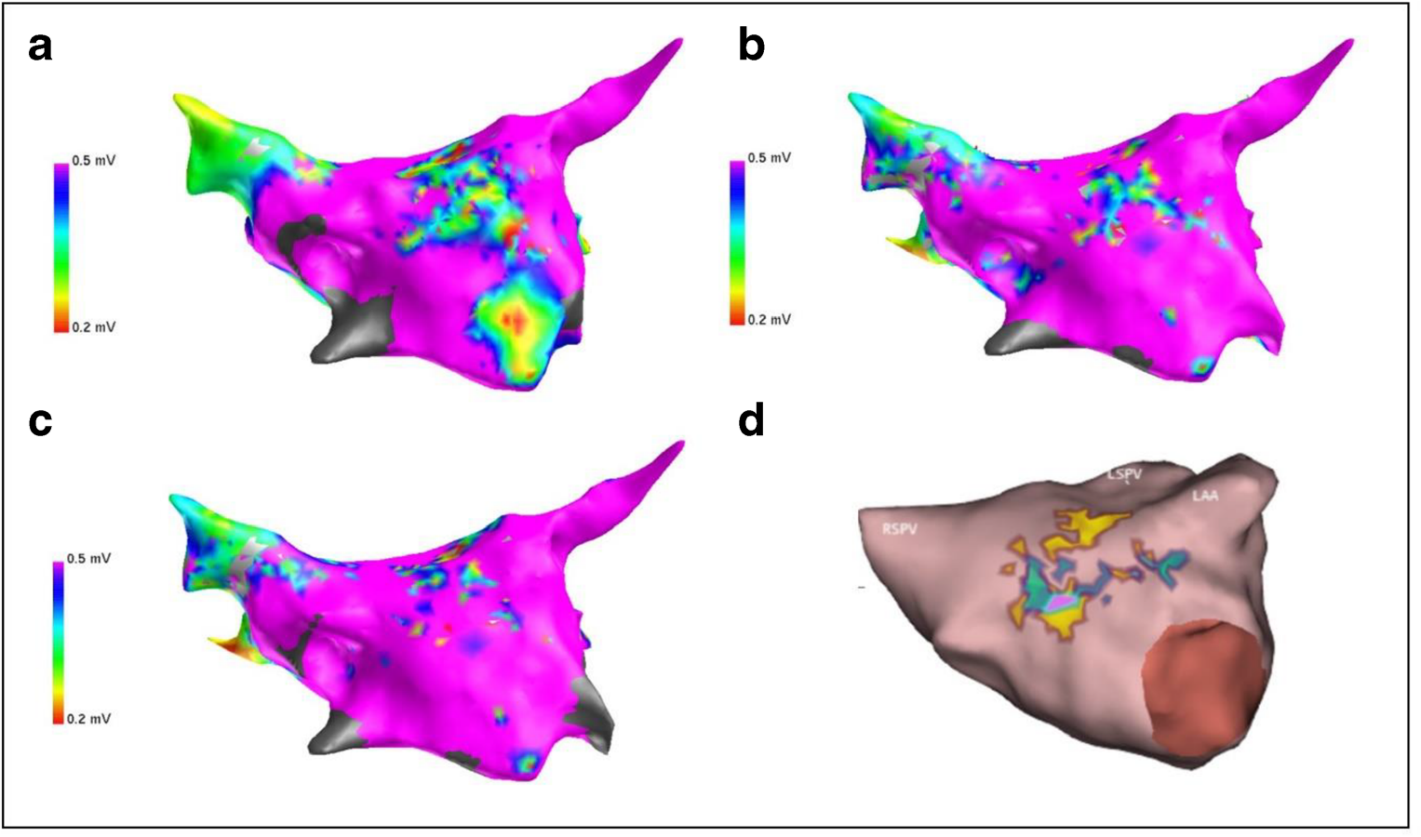
LA Anatomy in AP view. **a**, **b**, and **c** represent bipolar voltage maps made in sinus rhythm, pacing coronary sinus distal and coronary sinus proximal at 600 ms respectively. **d** represents AcQTrack data where yellow, green and purple means high concurrencies of LIA, LRA, and Focal patterns respectively. LIA localized irregular activity; LAR localized rotational activation

In general, percentage of spatial overlap between LVAs and LCC-cores was poor. The percentage of spatial overlap between LVAs and LCC patterns detected by AcQTrack increased with increasing LVA cut-off values. LIA had a significantly higher overlap with LVA compared to LRA and Focal patterns when LVAs were defined by voltages equal to or higher than 0.5 mV, *p* ≤ 0.05 (Table 3 and Fig. 2).

**Table 3:** Spatial Overlap of LIA/LRA/Focal patterns determined by AcQTrack and with LVA determined by composite maps

LVA definition mV	Spatial overlap %	Spatial overlap %	Spatial overlap %	*p* values	*p* values	*p* values
	LIA with LVZ	LRA with LVZ	Focal with LVZ	LIA vs LRA	LIA vs F	LRA vs F
0.2	4.97 ± 7.39	3.27 ± 5.25	1.09 ± 1.92	0.173	0.101	0.139
0.3	7.21 ± 9.02	4.52 ± 6.54	1.91 ± 2.70	0.092	0.075	0.161
0.4	9.60 ± 10.51	6.06 ± 7.76	3.12 ± 3.72	0.054	0.060	0.175
0.5	12.59 ± 11.81	7.80 ± 9.20	4.62 ± 5.27	0.020	0.036	0.187
0.6	16.40 ± 12.92	10.30 ± 10.37	6.02 ± 5.98	0.009	0.012	0.103
0.7	19.87 ± 14.41	11.83 ± 11.47	7.34 ± 6.93	0.003	0.005	0.103
0.8	23.4 ± 15.75	13.53 ± 11.52	7.85 ± 7.05	0.003	0.002	0.056
0.9	26.94 ± 16.95	15.28 ± 11.37	9.76 ± 8.80	0.003	0.002	0.107
1.0	30.30 ± 18.67	17.01 ± 11.65	9.32 ± 6.98	0.004	0.001	0.023

*LVA* low-voltage areas; *LIA* localized irregular activity; *LAR* localized rotational activation

The average distance from the LCC-core to the border of the LVA, with cut-off value of 0.2 mV, was 28.41 ± 15.56 mm, 29.03 ± 19.57 mm, and 35.62 ± 19.78 mm for LIA, LRA and Focal LCC patterns, respectively. The average distances from the center of the LCC-core to the border of the LVA decreased with increasing cut-off for the LVA (Table 4). There was no significant difference between the average or minimum distances from the center of the LCC-cores to the border of the LVAs among the different LCC patterns.

**Table 4:** Average and Minimal distances measured from the core of the LIA, LRA and Foci to the border of the LVA

LVA definition (mV)	Average distance (mm)	Minimal distance (mm)	Average distance (mm)	Minimal distance (mm)	Average distance (mm)	Minimal distance (mm)
	LIA to center LVA	LIA to center LVA	LRA to center LVA	LRA to center LVA	F to center LVA	F to center LVA
0.2	28.41 ± 15.56	18.95 ± 16.64	29.03 ± 19.57	18.07 ± 13.86	35.62 ± 19.78	26.70 ± 19.44
0.3	23.98 ± 13.80	17.04 ± 14.27	26.01 ± 18.24	15.32 ± 13.44	33.47 ± 19.97	25.35 ± 19.16
0.4	23.33 ± 16.09	14.48 ± 15.09	24.29 ± 19.73	14.61 ± 14.28	30.68 ± 19.38	22.32 ± 18.54
0.5	19.54 ± 13.22	10.46 ± 13.75	17.87 ± 15.79	10.12 ± 13.36	26.26 ± 19.16	19.75 ± 19.52
0.6	15.03 ± 13.47	7.35 ± 13.59	13.85 ± 15.48	6.66 ± 13.37	21.81 ± 16.36	14.35 ± 19.24
0.7	14.52 ± 13.22	5.09 ± 13.95	12.03 ± 13.60	4.58 ± 10.14	19.33 ± 13.32	10.12 ± 14.83
0.8	12.49 ± 13.87	3.39 ± 12.74	9.18 ± 14.05	3.64 ± 10.88	16.92 ± 13.02	7.89 ± 14.25
0.9	11.43 ± 12.20	1.75 ± 12.12	5.18 ± 12.06	1.21 ± 9.22	11.35 ± 12.03	4.65 ± 12.49
1	6.75 ± 10.24	− 0.44 ± 9.75	4.12 ± 11.40	0.19 ± 7.41	10.12 ± 10.04	2.79 ± 9.97

*LVA* low-voltage areas; *LIA* localized irregular activity; *LAR* localized rotational activation

**Fig. 3:**
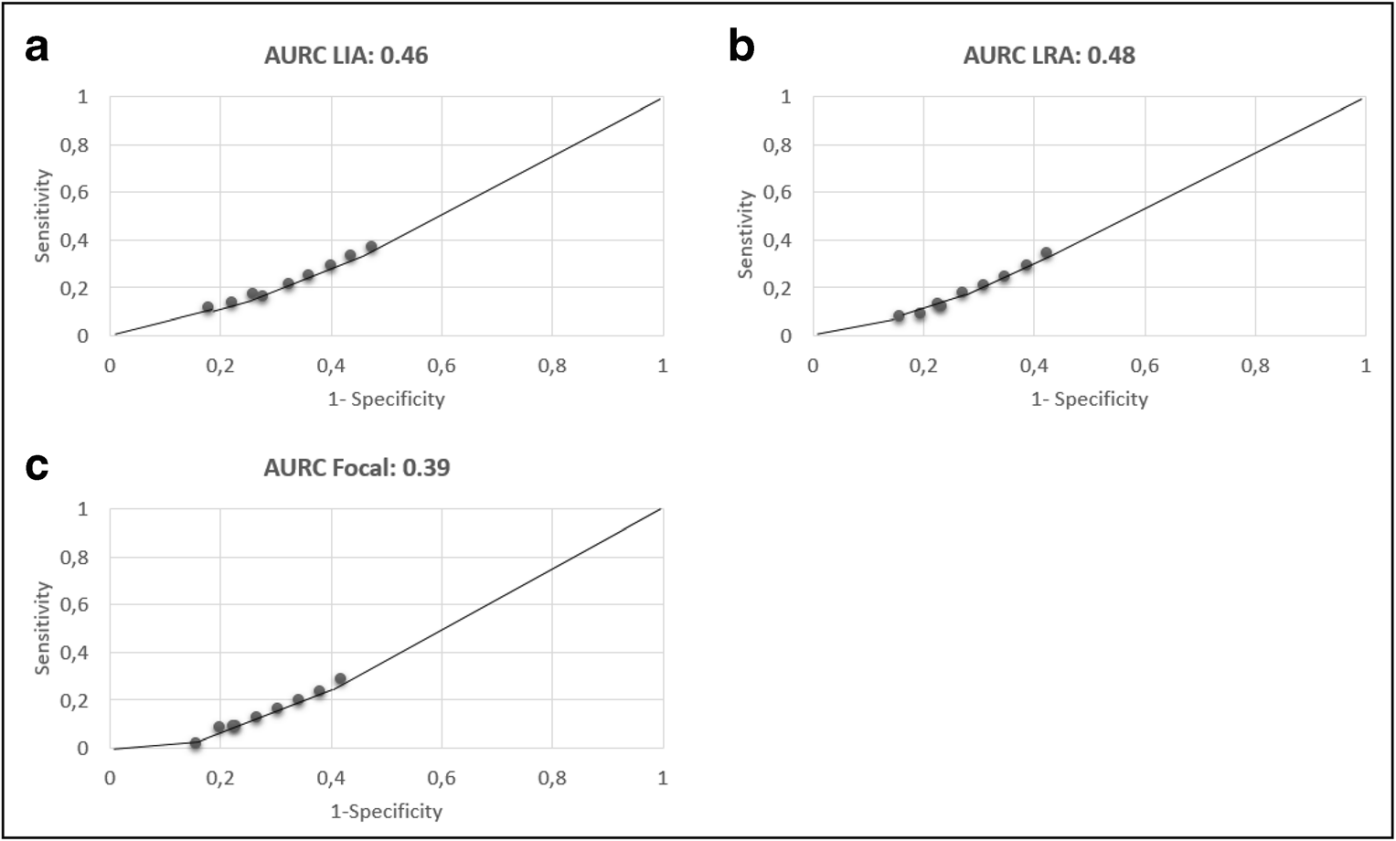
**a**, **b**, and **c** represent Area Under the curve (AURC) for LIA, LRA, and Focal patterns respectively. LIA localized irregular activity; LAR localized rotational activation

Sensitivity and specificity were calculated for PBP analysis on all LVA composite maps. LVA showed low sensitivity and high specificity for LIA, LRA, and Focal patterns detected by AcQTrack. Sensitivity and specificity are not significantly different when comparing composite maps with different LVA cut-offs (Table 5). AURC was 0.46, 0.48, and 0.39 for LIA, LRA, and Focal, respectively (Fig. 3). Cohen’s kappa coefficient was − 0.06 ± 0.01, − 0.08 ± 0.01, and − 0.08 ± 0.01 for LIA, LRA, and Focal patterns, respectively (Table 6).

**Table 5:** Sensitivity and specificity calculated based on point-by-point analysis comparing LIA/LRA/Focal with LVA determined by composite maps

LVA definition mV	Sensitivity LIA	Specificity LIA	Sensitivity LRA	Specificity LRA	Sensitivity Focal	Specificity Focal
0.2	0.12 ± 0.13	0.82 ± 0.17	0.08 ± 0.08	0.84 ± 0.15	0.02 ± 0.02	0.84 ± 0.15
0.3	0.13 ± 0.12	0.78 ± 0.21	0.09 ± 0.08	0.80 ± 0.19	0.08 ± 0.16	0.80 ± 0.18
0.4	0.16 ± 0.15	0.72 ± 0.23	0.12 ± 0.14	0.77 ± 0.20	0.09 ± 0.12	0.77 ± 0.20
0.5	0.17 ± 0.14	0.74 ± 0.14	0.13 ± 0.14	0.77 ± 0.14	0.09 ± 0.10	0.78 ± 0.15
0.6	0.21 ± 0.15	0.68 ± 0.17	0.17 ± 0.15	0.73 ± 0.14	0.12 ± 0.13	0.73 ± 0.15
0.7	0.25 ± 0.16	0.64 ± 0.17	0.21 ± 0.17	0.69 ± 0.14	0.16 ± 0.16	0.70 ± 0.15
0.8	0.29 ± 0.17	0.60 ± 0.18	0.25 ± 0.18	0.65 ± 0.14	0.19 ± 0.18	0.66 ± 0.15
0.9	0.33 ± 0.17	0.56 ± 0.18	0.29 ± 0.19	0.61 ± 0.15	0.24 ± 0.19	0.62 ± 0.16
1	0.37 ± 0.18	0.53 ± 0.17	0.34 ± 0.19	0.58 ± 0.15	0.28 ± 0.20	0.58 ± 0.16

*LVA* low-voltage areas; *LIA* localized irregular activity; *LAR* localized rotational activation

**Table 6:** Cohen’s kappa coefficient

LVA definition (mV)	LIA	LRA	Focal
0.2	− 0.0366	− 0.05664	− 0.08127
0.3	− 0.04413	− 0.0677	− 0.09232
0.4	− 0.05782	− 0.08923	− 0.10911
0.5	− 0.05631	− 0.07669	− 0.09161
0.6	− 0.06555	− 0.07962	− 0.0883
0.7	− 0.07202	− 0.08868	− 0.07489
0.8	− 0.07335	− 0.08984	− 0.0736
0.9	− 0.07096	− 0.08464	− 0.07046
1.0	− 0.06011	− 0.07503	− 0.08325
Average	− 0.06	− 0.08	− 0.08
STDEV	0.01	0.01	0.01

Determined to assess the inter-rater reliability between LVA detected by bipolar voltage mapping and LIA, LRA, and Focal complex conduction patterns during atrial fibrillation. *LVA* low-voltage areas; *LIA* localized irregular activity; *LAR* localized rotational activation; *STDEV* standard deviation

### Follow-up

After a mean 16 ± 5.56 months, 6 patients (60%) did not experience recurrence of any atrial arrhythmia. Ablation consisted in the creation of a posterior box, and mitral isthmus line.

## Discussion

This study is the first to investigate the relationship between LVA and LCC patterns involved in initiating and maintaining PsAF. The main findings of this study are as follows:

- LVA identified with BVM only partially overlap when the directionality of the wave front is altered.
- LCC pattern-cores observed during PsAF only partially co-localize with LVAs.

### Bipolar amplitude maps

BVM has emerged as an invasive tool for characterization of arrhythmogenic substrate and for putative guidance of endocardial ablation in PsAF [10]. LVAs determined with BVM are considered markers for fibrotic tissue involved in initiating and maintaining PsAF. Although BVM is regarded as the gold standard for substrate mapping, it is subjective on multiple factors [8]. Besides the known influences of electrode size, spacing, and degree-of-contact with the tissue, it is also heavily dependent on the directionality and rate of the wave front approaching the electrode [11]. The latter mentioned factors vary the arrival time of the activating wave front at each electrode, thereby altering the amplitude and morphology of the bipolar signals. These effects of so-called bipolar-blindness can impose significant challenges on detection of LVA with BVM and reduce the specificity of discrimination of the fibrotic substrate. Furthermore, the threshold for defining a LVA has not yet been established against an unambiguous, histologically valid standard [12].

**Fig. 4:**
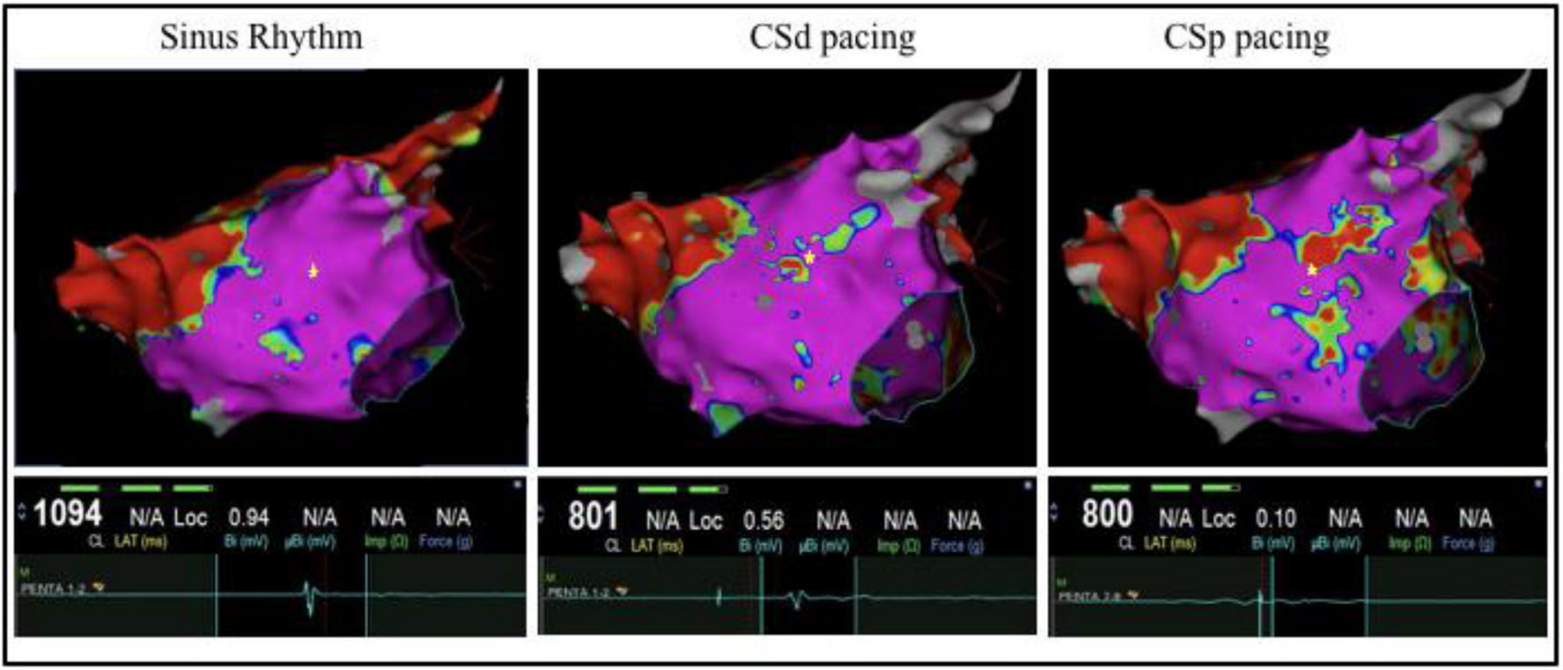
Directionality dependency of Bipolar EGM. High-definition CARTO maps acquired with the PentaRay during SR, CSd, and CSp pacing. Local EGM with respective bipolar amplitude (mV) are show from marked location on the map (yellow star)

The present study shows that when changing the direction of the wave front toward the bipolar electrode-pair, the average amplitude during SR is significantly higher as compared to pacing from the CS. These findings are in line with previous studies that reported a direction dependency in the amplitude of bipolar signals (Fig. 4) [13].

The present study also shows that the percentage of spatial overlap between LVAs identified from BVMs was low among the comparative map groups for both 0.2 and 0.5 mV cut-off values. This was true despite the observation that LVA as a percentage of total LA area did not significantly change with varying wave front directionality among the groups. The low percentage of spatial overlap is consistent with recent reports. Kalman et al. [14] reported that, in patients with PsAF, variation in rate and directionality of the wave front produces both general and regional changes in voltage, conduction velocity, and complex fractionation, resulting in significant changes in the BVM. Lim, et al. [15] described that the correlation between LVA and MRI-DE in the LA is significantly improved when acquired during PsAF vs SR. More specifically, they reported that, with adequate sampling, mean PsAF voltage is a reproducible marker reflecting the functional response to the underlying PsAF substrate. This represents a limitation of our study. In fact we did not perform any mapping with the 3D sequential mapping system in AF. Also, in the left ventricle, Tung et al. [16] assessed the direction dependency of BVM to characterize scar in the setting of substrate mapping in patient with scar-mediated VT. They observed that differences in BVM are frequently seen by varying the wave front directionality of ventricular activation, with lower concordance in heterogeneous scar, as compared to dense scar.

### Localized complex conduction patterns and low-voltage areas

During the UNCOVER post-market clinical study [17], distinct LCC patterns, as previously described by Chi et al. [9] in PsAF, were observed and categorized in all 129 subjects.

To the best of our knowledge, the relationship between these LCC pattern-cores and LVAs identified by BVM had not been investigated previously. As the individual BVM had a very low spatial-overlap, due to directional-dependency, we decided that the relationship between LCC pattern-cores and LVAs should be investigated on composite maps of BVM. We hypothesized that the composite maps of the individual SR and CSd and CSp pacing would be reflecting the LVA more accurately as we aggregate the amplitude from different directions of the wave fronts across the substrate. The present study shows that there is only a moderate percentage of spatial overlap between all three groups of LCC-cores and LVAs based on the composite maps. Furthermore, low sensitivity and high specificity suggest that LVA offers a poor predictive value to identify location of LCC-cores. The negative kappa coefficient reinforces this observation.

The literature presently contains few studies where the relationship between activation mapping during PsAF and substrate mapping with late gadolinium-enhanced (LGE) cardiac magnetic resonance (MRI) or BVM was investigated.

A previous study by Cochet et al. [18] examined the relationship between reentrant-driver mechanisms, determined with ECGI, and LGE-MRI. The study concluded that reentrant drivers in human PsAF are partially fibrosis mediated. Another study by the same group confirmed this, as they observed a clustering of the same re-entrant driver activity at LGE borders [19].

Deneke et al. [20] investigated the spatial relationship between focal impulses and rotors sources in PsAF with LVA. They reported a wide discrepancy in the spatial distribution of LVA determined with BVM and the location of foci and rotors as identified with FIRM mapping.

Another study highlighted that CFAE, which are associated with slow anisotropic conduction, and might be involved in the maintaining and perpetuation of PsAF, are not co-localized with LGE regions of interest. However, CFAE might also be a result from far-field signals arising from adjacent myocardium or wave collision during PsAF or rapid focal or re-entrant activity [21]. The results of these previous studies are in line with the findings of the present study, where we describe a poor predictive value of LVA for the localization of LCC-cores during PsAF. Nevertheless, we acknowledge the differences in substrate characterization and activation mapping performed in the aforementioned studies and in the current study.

## Clinical implications

Wave front direction-dependent discrepancies observed in the LVAs identified from BVM imply that guidance of ablation strategies in the treatment of patient with PsAF is questionable with this method of substrate characterization. Importantly, the largest discrepancies appear to lie within the most heterogeneous substrate, which are also more likely to be involved in initiating and maintaining PsAF. Furthermore, LCC pattern-cores observed during PsAF are only partially co-localized with LVAs identified with BVM. This significantly limits the usage of a LVA acquired from a single, unidirectional wave front as a surrogate for substrate mapping to identify clinically relevant LCC-cores in PsAF.

## Conclusion

Due to wave front direction dependency, LVAs mapped with BVM in SR and during CS pacing only partially overlap in patients with PsAF. LCC-cores mapped during PsAF partially co-localize with LVAs.

## Limitations

The present study has a small patient population; however, the large amount of collected points of data (approx. 3650 per patient for PBP comparison) partially mitigates this limitation. The present study demonstrates significant heterogeneity across the 10 patients, in percentage of overlap of LVAs across the map groups in the LA. Finally, as previously stated in the discussion, the fact that no mapping with the 3D CARTO mapping system was performed in AF should be considered an important limitation of our study.
